# Electro-chemical deposition of nano hydroxyapatite-zinc coating on titanium metal substrate

**DOI:** 10.1186/s40729-017-0095-1

**Published:** 2017-08-13

**Authors:** N. A. El-Wassefy, F. M. Reicha, N. S. Aref

**Affiliations:** 1 Dental Biomaterials Department, Faculty of Dentistry, Mansoura University, 35516 El Gomhoria St., Mansoura, Egypt; 2 Physics Department, Faculty of science, Mansoura University, 35516 El Gomhoria St., Mansoura, Egypt

**Keywords:** Hydroxyapatite-zinc coating, Titanium metal, Surface roughness, Surface morphology, Coating adhesion, Electrochemical deposition

## Abstract

**Background:**

Titanium is an inert metal that does not induce osteogenesis and has no antibacterial properties; it is proposed that hydroxyapatite coating can enhance its bioactivity, while zinc can contribute to antibacterial properties and improve osseointegration.

**Aims:**

A nano-sized hydroxyapatite-zinc coating was deposited on commercially pure titanium using an electro-chemical process, in order to increase its surface roughness and enhance adhesion properties.

**Methods:**

The hydroxyapatite-zinc coating was attained using an electro-chemical deposition in a solution composed of a naturally derived calcium carbonate, di-ammonium hydrogen phosphate, with a pure zinc metal as the anode and titanium as the cathode. The applied voltage was −2.5 for 2 h at a temperature of 85 °C. The resultant coating was characterized for its surface morphology and chemical composition using a scanning electron microscope (SEM), energy dispersive x-ray spectroscope (EDS), and Fourier transform infrared (FT-IR) spectrometer. The coated specimens were also evaluated for their surface roughness and adhesion quality.

**Results:**

Hydroxyapatite-zinc coating had shown rosette-shaped, homogenous structure with nano-size distribution, as confirmed by SEM analysis. FT-IR and EDS proved that coatings are composed of hydroxyapatite (HA) and zinc. The surface roughness assessment revealed that the coating procedure had significantly increased average roughness (Ra) than the control, while the adhesive tape test demonstrated a high-quality adhesive coat with no laceration on tape removal.

**Conclusions:**

The developed in vitro electro-chemical method can be employed for the deposition of an even thickness of nano HA-Zn adhered coatings on titanium substrate and increases its surface roughness significantly.

## Background

Titanium metal is one of the most widely used biomedical orthopedic materials because of its decent mechanical properties [1]. However, as an inert material﻿, it cannot induce osteogenesis and has no antibacterial properties [2]. In order to improve surface bioactivity of titanium substrates, numerous methods have been proposed to cover it with bio-ceramic coatings [1]. Various clinical studies demonstrated that the hydroxyapatite coating of prosthesis can promote earlier osseous response which could increase the prosthesis fixation and the bonding strength [3–5].

Titanium implants are usually placed in contact with bones and gingival tissues so they are partially exposed to the oral cavity during and after implantation. This increases the hazard of bacterial infection, which is known as peri-implantitis [6, 7].

For centuries, Zinc (Zn) as one of the essential elements of tissues in the human body has a stimulating role in the metabolism of bones a﻿nd has been used as bacteriostatic and bactericidal agents [8, 9]. Zinc can enhance the retention strength and osseointegration of implants [10, 11], by stimulating alkaline-phosphatase activity and collagen production, thus can increase bone deposition and reduce bone resorption [12]. Zn deficiency results in skeletal changes, including retardation of skeletal growth [10] and prolonged bone recovery [13]. Moreover, Zn species are also known to possess excellent antibacterial qualities. Zinc showed inhibitory effects against several bacteria, including *Streptococcal mutans* [14–16].

The metals’ antibacterial activity has been contingent on their contact surface; thus, a greater nanoparticles’ surface area permits larger interfaces and increases their interactions with other particles [17].

Although HA coatings revealed an enhanced bone attachment and thus better implants integration, long-term coating stability is quite a provoking concern [18]. Numerous coating techniques like plasma spraying, sol-gel, electrophoretic deposition, electro deposition have been employed to deposit hydroxyapatite on titanium implants. Plasma spraying is the most widely used technique for coating, but it leads to decomposition of HA due to the high temperature used, and it cannot be employed for complex structures. In electrophoretic deposition, high voltage was applied to the metal surface in order to attract the dispersed particles which leads to anodic polarization of metal substrate. This might increase the corrosion risk of metal and suppress the adhesion of HA particles [19–21]. Electro-chemical deposition (ED) is a frequently used approach with increasing popularity, due to variability of coating composition, process simplicity, and its applicability for multidimensional implant surfaces [22].

The aim of the present work was to develop well-adhered and uniform hydroxyapatite-zinc coatings on titanium metal substrate, through an in vitro electro-chemical deposition method. The coating was characterized for functional chemical group, surface morphology, surface chemical analysis, surface roughness, and coat adhesive bonding by Fourier transform infrared spectrometer (FT-IR), scanning electron microscope (SEM), energy dispersive spectroscope (EDS), profilometer, and tape adhesive test respectively.

## Methods

### Cathode preparation

Commercially pure Ti (CpTi) grade II specimens were cut down into plates with dimensions 10 × 10 × 2 mm and used as substrates (cathode material) for depositing HA and Zn. CpTi specimens were polished with successive grades of silicon carbide papers, ultra-sonicated in acetone (99.5%, EM Science), rinsed in distilled water, and then air dried at room temperature, before they were used for the electro-chemical process.

### Electro-chemical deposition of HA and Zn

**Fig. 1:**
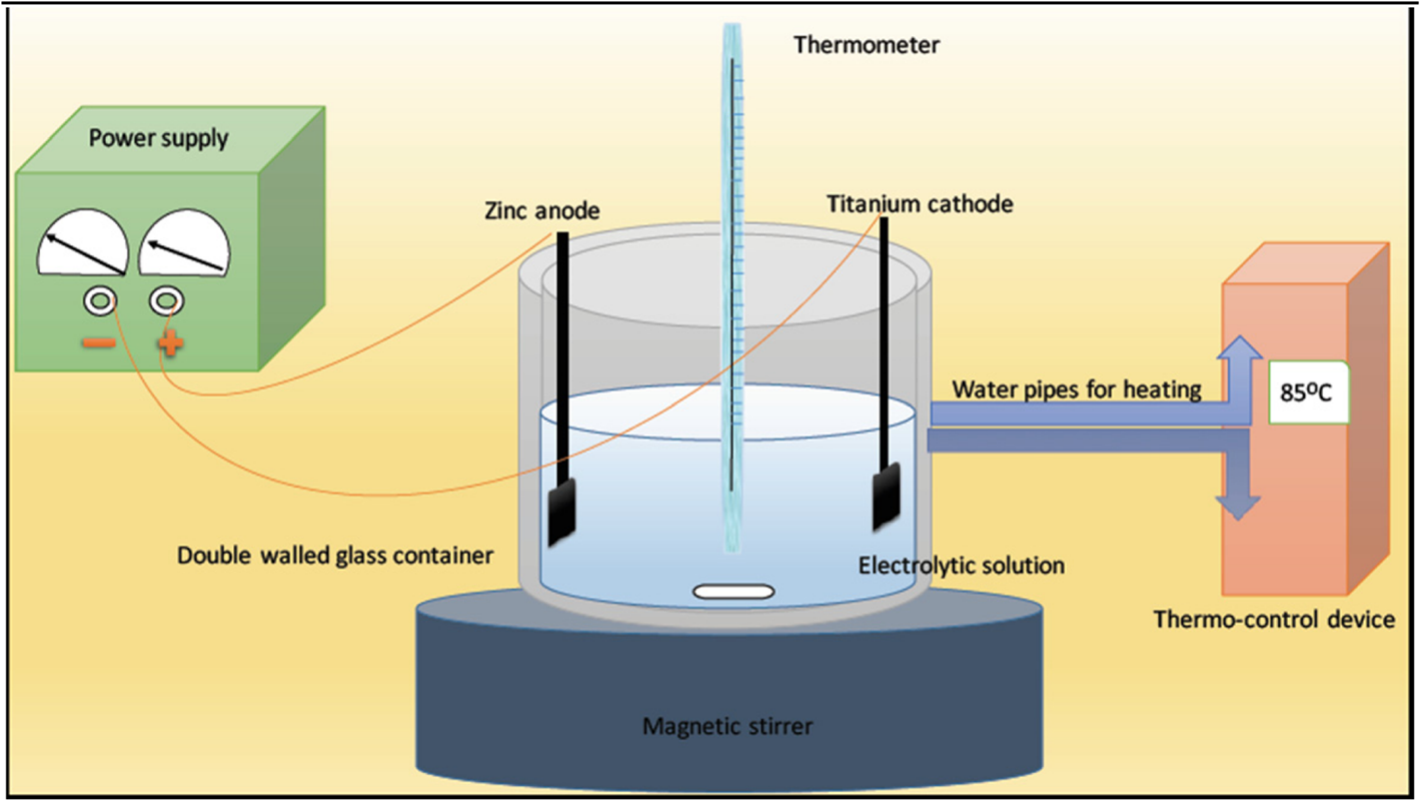
Graphical presentation of the electrochemical-deposition coating process’ equipment

The electro-chemical deposition process was carried in an electrolytic solution. The Ca source of the electrolyte was prepared from dry cuttlebone (CB) (Sepia officinalis L., from the Mediterranean Sea). The CB was cut into blocks and immersed into 5% household bleach NaClO for 2 days, in order to eliminate the organic component [23], then rinsed with water and dried in an oven at 80 °C for 6 h. The starting CaCO_3_ material of CB was made to react with nitric acid 69% (SD F﻿ine Chem Limited, India) to form Ca(NO_3_)_2_ solution in water. After the complete evolution of CO_2_, water was evaporated by heating and the resultant powder was examined by FT-IR spectrometer (Nicolet iS10, Thermo Electron Corporation, UK) which utilized the selected range of 400 to 4000 wave numbers (cm^−1^) to confirm its chemical structure of Ca(NO_3_)_2_·4H_2_O. The other salts were purchased from Sigma Aldrich and added to form an electrolytic solution containing 0.6 M Ca(NO_3_)_2_·4H_2_O, 0.36 M(NH_4_)·2HPO_4_, 1 M NaNO_2_, 6% H_2_O_2_, and NH_4_OH ﻿to adjust the solution ﻿pH﻿ to 6. Pure zinc (Zn) particles (Zinc Tres Pur, Prolabo, N 29050, *N* = 99.999) were pressed in a bench press; Craver Laboratory Press (Model C 31000-823, USA) to produce a 10 × 10 × 2mm plates that acted as the anode. Platinum wires were used to hang the electrodes in the solution. A thermometer was used to monitor the temperature during the process. The deposition process was carried out with a power supply unit (LT ECOS, 7972, Italy) by applying an electrode potential of ~2.5 V at 85 °C temperature stabilized by a thermostatic water bath (MLW, U4, 74010, Germany) for 2 h, during the deposition process a continuous stirring was carried out by a magnetic stirrer. The electro-chemical deposition setup is shown in the schematic diagram in Fig. 1. After the deposition, specimens were taken out from the electrolytic bath, rinsed with deionized water, and left to dry for 24 h on a clean bench. The coated CpTi specimens were then sintered at 400 °C for 2 h in an electric furnace ﻿with a heating rate of 5 °C/min and gradually cooled to room temperature inside the furnace.

### Characterization of the deposited HA-Zn coating

#### Infrared analysis

The coating was scrapped from Ti specimen's surface and investigated for its chemical structure using FT-IR spectroscopy. The powder was investigated by double-beam dispersive IR spectrometer (Nicolet iS10, Thermo Electron Corporation, UK) which utilized the selected range of 400 to 4000 wave numbers (cm^−1^) at 4 cm^−1^ resolution and averaging of 100 scans. Two milligrams of scrapped powder was mixed with 300 mg of KBr and pressed into a disc before the measurement.

#### Surface characterization of coatings

Scanning electron microscope (SEM) (JSM 6300, JEOL, Japan) and energy dispersive spectroscope ﻿(EDS) were used to examine the morphological qualities and the elemental composition of the HA-Zn deposits. The working distance was 15 mm at 20 V. Three specimens were examined for each group of the study.

#### Surface-mechanical testing of coatings

##### Roughness of coatings

Specimens of control and HA-Zn coated groups were evaluated by a surface roughness profilometer tester (Surftest SJ-210, Mitutoyo Corporation, Tokyo, Japan,) according to ISO 4287-1997 [24] with ﻿a diamond tip radius of 5 μm, ﻿a scanning speed 0.5 mm/s, a resolution of 0.01 μm, a Gaussian filter, and a cut-off length of 8 mm. Seven specimens from each group were scanned and evaluated for the average roughness parameter, each specimen was scanned five times, and the mean was calculated in µm. The roughness parameter (Ra) values were compared for statistical significance using the Student *t* test in SPSS software version 20 (SPSS Inc. Chicago, IL, USA).

##### Coating adhesion test

The adhesion of coating is qualitatively assessed by the tape test. A standard test method (Tape test-ASTM D 3359-97) was used for assessing the adhesion of the HA-Zn coating on the titanium substrate. In this method, a part of a pressure-sensitive adhesive tape (masking tape, M&G pen AJD97355) is pressed against the coating by the use of a pencil eraser for 90 s. The tape is then rapidly removed (without jerk movements) at 180° angle, and the degree of film removal is detected when the tape is pulled off. Because an integral coating with substantial adhesion is often not detached at all, the sternness of the test is typically improved by making a figure X cutting into the coat using a sharp scalpel with enough pressure to reach the metal substrate, then applying the tape and remove it. The denuded area is inspected for removal of coating from the substrate, and then the adhesion is ranked by relating the detached part of the coat versus a recognized rating scale. The test is repeated for three other locations in the same specimen. Coverage of coated substrate was computed using Matlab (version7.1) [25].

## Results

### FT-IR results

**Fig. 2:**
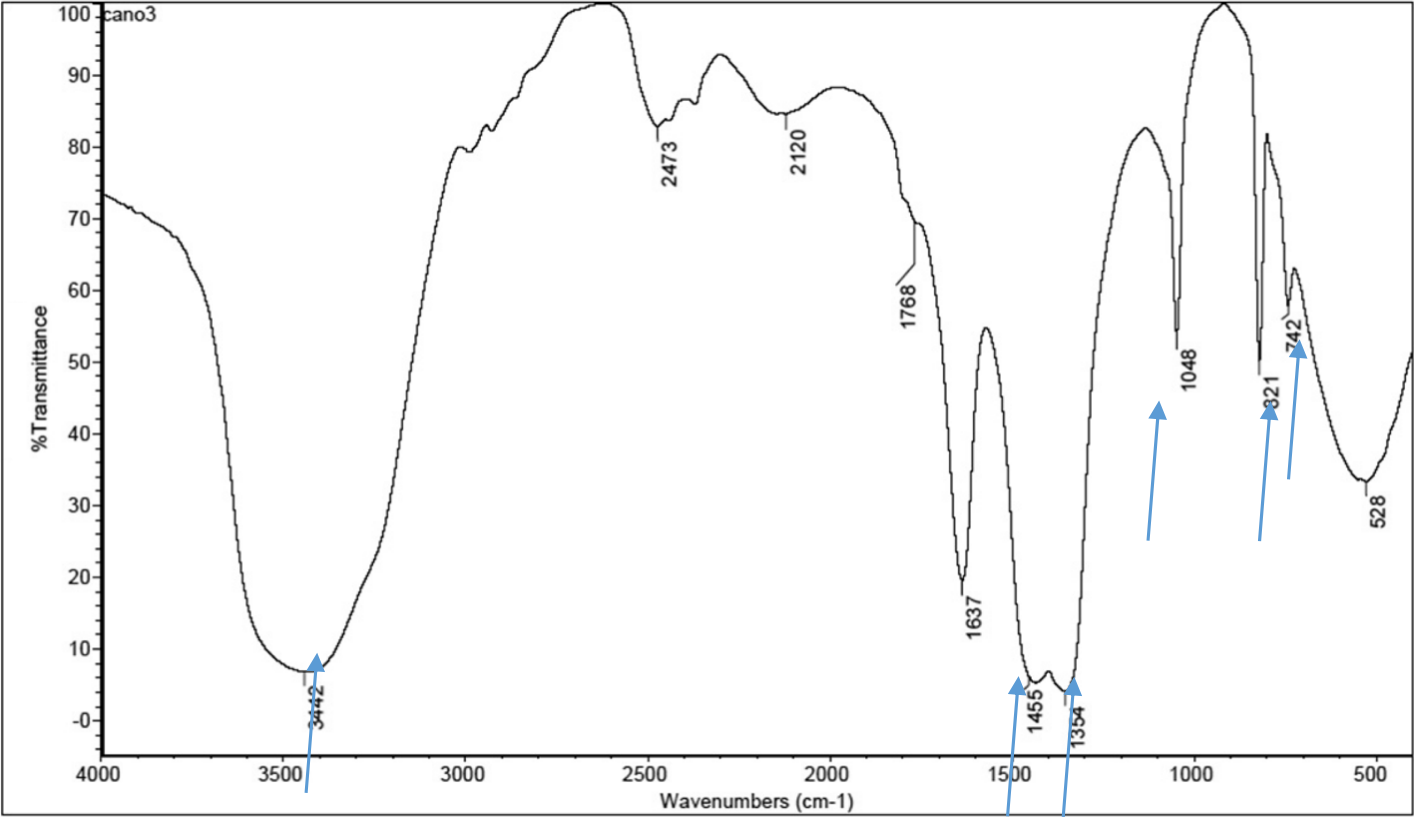
IR spectra of Ca(NO_3_)_2_·4 H_2_O powder prepared from a natural source (CB)

**Fig. 3:**
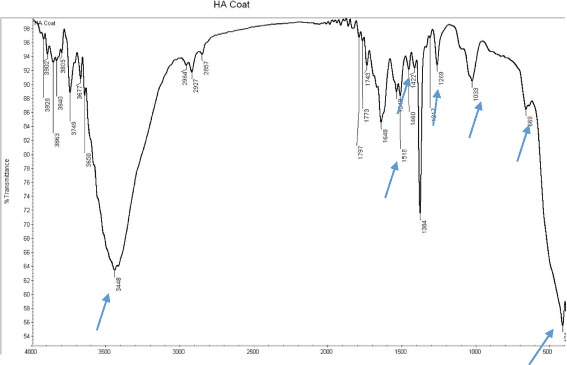
IR spectra of HA-Zn powder scrapped from coated titanium specimen

Figure 2 shows the FT-IR spectra of Ca(NO_3_)_2_·4H_2_O with weak sharp absorption peak bands at 742, 821, and 1048 cm^−1^, a strong broad absorption band at 1354 cm^-1^, and a strong shoulder absorption band at 1455 cm^−1^. A wide broad absorption band peak appears at 3442 cm^−1^ due to the presence of water. Figure 3 shows the FT-IR spectra of HA-Zn powder scrapped from CpTi specimens; the band at ~421 cm^−1^ may be due to the stretching vibrational mode of Zn–O. The absorption bands at 669 cm^−1^ and around 3448 cm^−1^ are due to the stretching vibration and the bending of the O–H bond that contributes in hydrogen bond formation between water molecules adsorbed by hydroxyapatite and potassium bromide used for pellet preparation. The wide broad absorption band in the area of 1033–1269 cm^-1^ wave number is attributed to the stretching vibrations of P–O bonds in the phosphate group (PO_3_^−4^). The low-intensity band presented in the area of 1422–1518 cm^−1^ wave numbers is attributed to the stretching vibration of the C–O bond of the carbonates (CO_3_^−2^).

### SEM and EDS results

**Fig. 4:**
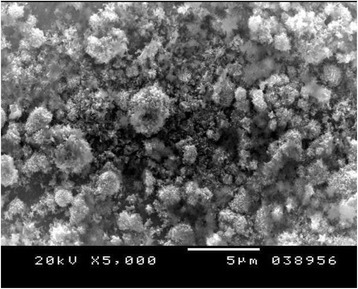
Scanning electron microphotograph of Cp titanium specimen coated with nano HA- Zn at ×5000

**Fig. 5:**
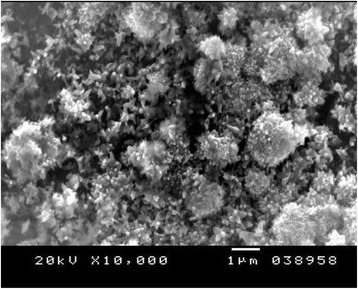
Scanning electron microphotograph of Cp Titanium specimen coated with HA-Zn at X10,000

**Fig. 6:**
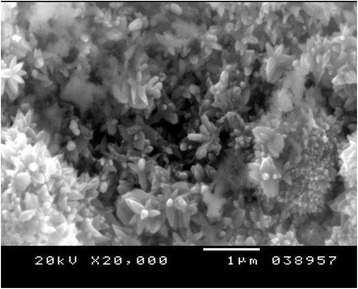
Scanning electron microphotograph of Cp titanium specimen coated with HA-Zn at ×20,000

**Fig. 7:**
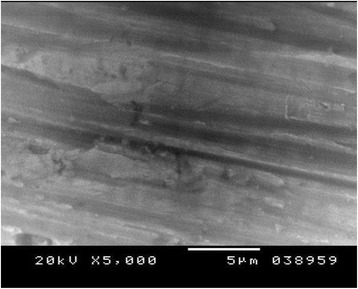
Scanning electron microphotograph of control Cp Titanium specimen at X 5,000

**Fig. 8:**
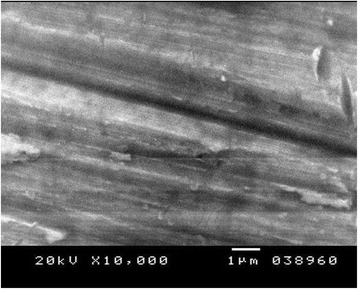
Scanning electron microphotograph of control Cp titanium specimen at ×10,000

**Fig. 9:**
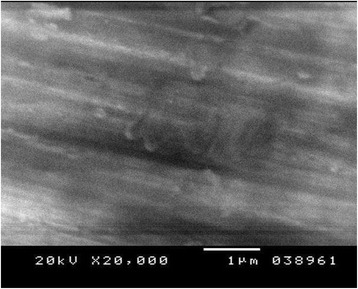
Scanning electron microphotograph of control Cp titanium specimen at ×20,000

**Fig. 10:**
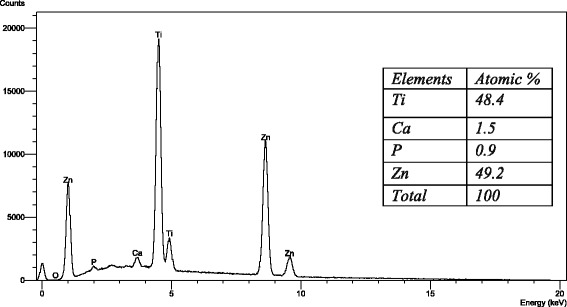
Energy dispersive spectrum of Cp titanium specimen coated with HA-Zn

**Fig. 11:**
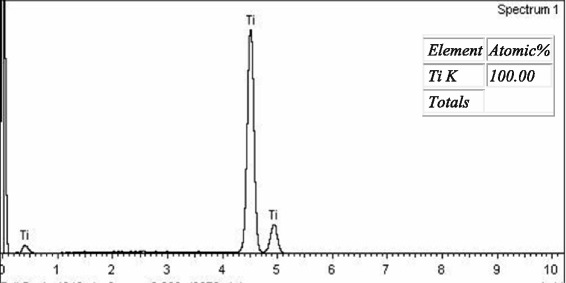
Energy dispersive spectrum of control Cp titanium specimen

Figures 4, 5, and 6 show the SE microphotographs of CpTi specimens coated with HA-Zn, the specimens’ surface is homogenously covered with evenly distributed globular/rosette-like nano-structures that tend to aggregate in characteristic cluster forms with littl﻿e intervening porosity. On the other hand, Figs. 7, 8, and 9 show the SE microphotographs of control CpTi specimens with blanc surfaces; only the cutting lines of machining appear. The EDS analysis of HA-Zn shows the presence of zinc, titanium, calcium, and phosphorus; the atomic ratio of Ca/P is 1.67 (Fig. 10). However, the control specimen only contains titanium element (Fig. 11).

### Roughness results

Table 1 shows the mean average roughness value of the HA-Zn coated and control specimens; the average roughness is 0.34 μm for the control group and increased significantly to be 1.09 μm for the HA-Zn-coated group (*P* = 0.009) when compared using a pair comparison of the Student *t* test using SPSS version 20.

**Table 1:** The Student *t* test of the control and coated specimen roughness Ra (μm)

	Number of specimens	Mean ± (SD)	Standard error mean	*F* value	*P* value
Control	7	0.34 ± (0.06)	0.02	9.67	0.009*
HA/Zn coated	7	1.09 ± (0.16)	0.60		

### Adhesive test results

Following the examination of X cut areas after the adhesive tape removal; the adhesion was rated to be 5A, as no peeling or coat removal occurred along the incisions' length or at their intersection.

## Discussion

Metallic orthopedic prosthesis is most commonly used due to its good mechanical properties, but its failure mostly occurs due to the lack of proper bone bonding and/or the occurrence of post-operative infections. Hydroxyapatite is commonly used as a bone filler biomaterial or as a coat for titanium prosthesis due to its decent biocompatibility, osseoconductivity, and bioactivity [26]. However, as a ceramic material, HA still has lower mechanical properties [27]. The biological apatite differs from synthetic apatite because the former contains numerous cationic substitutions, such as Zn^2+^, Na^+^, Mg^2+^, and has smaller size than synthetic apatite [28, 29]. It was proposed that the addition of zinc to hydroxyapatite had led to a reduction in inflammatory reaction and an improvement of bioactivity [28, 30].

Plasma spraying, sol-gel, and electrophoretic deposition has been all utilized to deposit HA on titanium implants, with some difficulties and worries of suppressing the HA particles’ adhesion, anodic polarization of metal substrate, and increasing metals’ corrosion risk [19–21]. Electrochemical deposition (ED) is the selected approach in this study due to its simplicity, easiness of parameters control, uniform coating thickness produced, and its applicability for multidimensional implant surfaces [22].

In the current study, an electrochemical deposition was applied to prepare nano-HA-Zn coating on titanium metal aiming to improve bioactivity, osseointegration, and preventing peri-implantitis. At this early point of research, the coatings’ procedure was accustomed to produce a uniform thickness of HA-Zn coating, characterize its chemical structure, observe its surface morphology, and evaluate the surface roughness and coat adhesive properties.

Recycling of natural-derived resources is a challenging task that may have both environmental and economical profits. Cuttlebone fishery is a naturally derived biomaterial that was used as a source of calcium during the electrochemical deposition process in this study. It was confirmed in the IR spectra (Fig. 2) that Ca(NO_3_)_2_·4H_2_O resulted from the reaction of CaCO_3_ of cuttlebone and nitric acid [31]. The selected time for electrochemical deposition of HA-Zn coating was 2 h; as by then, the formation of a white detectable coating had occurred and could be scrapped for IR spectral analysis. After preparation of HA-Zn coating, the analyzed powder appeared to still have the HA characterization. Li et al. prepared Zn-HA coatings through a hydrothermal method and found that the FT-IR spectra of Zn-HA has no significant changes than the as-prepared HA [32]; this Zn-HA spectrum paralleled with this study.

Yang et al. prepared a Zn-HA coating on Ti plates by an electrochemical process, and the SEM examination showed irregularly shaped rod-like crystals with hexagonal cross-section; this corresponded well with the current study results. They also concluded that a Zn-HA coating improves proliferation and differentiation of osteoblasts and would enhance implant osseointegration [11].

Ceramic coatings must have good adhesion to the implant to act as a barrier and assure good protection to the substrate. The adhesion test was performed in this study to verify the adequacy of the coating thickness.

An improvement of coating adhesion occurs as their thickness decrease, although very thin coatings may not attain the protection requirements [33]. Contrariwise, it is recognized that thick ceramic coatings may develop cracks after the deposition procedure [34]. The adhesive tape test read the highest score (5A); this might be attributed to the fine homogenous, closely packed, coating particles that appear crack free and highly sintered, as proved by the SEM results in Figs. 4, 5, and 6.

Dental implants do exist with various geometries, different lengths and diameters, and features, such as, pits, pores, vents, and slots. Essentially, a highly rough surface produces better initial stability and anchorage. Moreover, a rough surface with a larger surface area facilitates particles exchange between the implant and surrounding tissues. It could be concluded that such coatings with an increased surface area could have better clinical performance [35]. This developed electro-deposition process, can be applied  to deposit a nano-HA-Zr coating to complex implant surfaces and thus increases their surface area, surface roughness, initial stability and clinical performance. 

Supplementary, biocompatibility, anti-bacterial activity﻿, and in vivo investigations are required to correlate between the HA-Zn coating properties and their effect on bone formation and osseo-integration.

## Conclusions

The electro-chemical method can be employed for HA-Zn coating deposition on titanium metal, where Ca source was a recycled cuttlebone fish to precipitate HA phases. Using a Zn anode on a low-sustained voltage was able to induce an even coat thickness of HA-Zn precipitation and increase the surface roughness significantly.
